# Notch signaling in cancer: metabolic reprogramming and therapeutic implications

**DOI:** 10.3389/fimmu.2025.1656370

**Published:** 2025-09-19

**Authors:** Shuang-Shuang Wang, Hui-Lin Lv, Rong-Zu Nie, Ya-Ping Liu, Yan-Jie Hou, Chen Chen, Xing-Yue Tao, Huo-Min Luo, Pei-Feng Li

**Affiliations:** ^1^ College of Food and Bioengineering, Zhengzhou University of Light Industry, Zhengzhou, China; ^2^ Henan Key Laboratory of Cold Chain Food Quality and Safety Control, Zhengzhou University of Light Industry, Zhengzhou, China; ^3^ Institute of Life and Health, Zhengzhou University of Light Industry, Zhengzhou, China

**Keywords:** notch signaling pathway, cancer, metabolic reprogramming, tumormicroenvironment, metabolism

## Abstract

The evolutionarily conserved Notch signaling pathway is essential for cell-fate determination, organogenesis, and tissue homeostasis. Notch receptors and their ligands are transmembrane proteins with epidermal growth factor–like repeats; ligand–receptor binding triggers canonical Notch signaling. Notch signaling is context dependent in cancer, functioning as either an oncogene or a tumor suppressor. Aberrant Notch activation promotes epithelial–mesenchymal transition, sustains cancer stem–like phenotypes, and drives metabolic reprogramming, thereby facilitating tumor progression and therapeutic resistance. Current clinical efforts target the pathway with γ-secretase inhibitors (GSIs), monoclonal and bispecific antibodies, and synthetic Notch (synNotch) approaches. Clinical translation, however, is constrained by dose-limiting toxicity, a paucity of predictive biomarkers, and compensatory resistance through intersecting pathways. Priorities for future work include the development of highly selective Notch modulators, biomarker-guided combination regimens, and targeted delivery systems to realize the translational potential of Notch-targeted therapies in precision oncology.

## 1 Introduction

Cancer has become a significant threat to global health, with a disease burden that cannot be ignored. In 2022, cancer resulted in 9.67 million deaths worldwide and accounted for 250 million disability-adjusted life years (DALYs). This significantly diminishes patients’ quality of life and leads to substantial social productivity losses (1). Moreover, the economic impact of cancer is also considerable; research predicts that from 2020 to 2050, cancer will cause economic losses of up to 25 trillion US dollars, accounting for 0.55% of the global gross product. Cancer presents a significant challenge to China’s healthcare system. In 2021, malignant tumors were the second leading cause of death for urban residents (24.61%) and the third for rural residents (22.47%). In 2022, China reported 4.77 million new cancer cases and 2.56 million cancer deaths, accounting for 25.48% of global cases and 26.47% of global deaths, respectively. Although China accounts for only 18% of the world’s population, it bears a disproportionately larger share of the global cancer burden, underscoring the significant challenges the country faces in cancer prevention and control.

In modern medicine, cancer remains a global health challenge, and its complexity and diversity necessitate a comprehensive understanding of its biological characteristics from multiple perspectives. Cancer reflects not only dysregulated cell proliferation but also extensive reprogramming of cellular metabolism, which furnishes the energy and biosynthetic precursors necessary for tumor growth and survival. The Notch signaling pathway has recently gained prominence in cancer metabolism research as a crucial factor in determining cell fate.

The Notch signaling pathway facilitates intercellular communication via receptor-ligand interactions, impacting cell differentiation, proliferation, and apoptosis. This pathway’s abnormal activation is linked to the onset and development of diverse tumors, where its influence can be either promotive or suppressive, contingent on the cell type and microenvironment. The Notch signaling pathway’s complexity stems from its capacity to perform varied functions across different biological contexts, highlighting its potential as a therapeutic target in cancer treatment.

Cancer metabolic reprogramming involves tumor cells altering metabolic pathways to support rapid proliferation, enhancing energy supply and biosynthetic precursor production. The Notch signaling pathway significantly influences tumor growth, survival, and therapeutic response by regulating essential metabolic pathways, including glucose, fatty acids, and amino acids. Current research focuses on the Notch signaling pathway’s role in glycolysis and its effects on fatty acid and amino acid metabolism. The Notch signaling pathway’s role in the tumor microenvironment is gaining increased attention. These interactions are vital for tumor initiation and progression, influencing tumor development and treatment responses, which are key areas for future research.

With a deeper understanding of the Notch signaling pathway’s role in cancer metabolism, targeted therapies are being developed and show promise. Future research will evaluate the efficacy and safety of these targeted treatments and investigate their synergistic effects with other therapies. Future research will concentrate on creating personalized treatment strategies tailored to the Notch signaling pathway’s activation status and patients’ unique genetic backgrounds.

In conclusion, elucidating the influence of Notch signaling on cancer metabolism holds considerable promise. It may reveal novel diagnostic biomarkers and therapeutic targets, improving treatment efficacy and patient quality of life. Continued research will facilitate the development of more precise, mechanism-based therapies and contribute to more effective management of this global health burden.

## 2 Overview of cancer and the notch pathway

### 2.1 Overview of cancer

Cancer (malignant neoplasm) is characterized by invasion of surrounding tissues and, often, dissemination to distant sites via the bloodstream and lymphatic system, resulting in metastatic tumors. Its development is a multistage, multifactorial process influenced by genetic, environmental, lifestyle, and other factors.

Cancer types vary and can be classified by tissue origin into epithelial-derived (e.g., lung, breast, and colorectal cancers) and non-epithelial-derived (e.g., lymphomas and leukemias). Cancer development generally results from genetic mutations in cells that can disrupt cell cycle regulation, inhibit apoptosis, promote angiogenesis, and enable immune evasion.

The clinical manifestations of cancer are diverse, and in the early stages, there may be no symptoms or the symptoms may be subtle. As the disease progresses, patients may experience symptoms such as lumps, pain, weight loss, and fatigue. Cancer treatment options encompass surgical resection, radiotherapy, chemotherapy, targeted therapy, and immunotherapy. Surgery is the preferred treatment method for many early-stage cancers, physically removing the tumor. Radiotherapy utilizes high-energy rays to damage cancer cell DNA, whereas chemotherapy uses drugs to inhibit cancer cell proliferation. Targeted therapy aims at specific molecular markers on cancer cells, while immunotherapy boosts the patient’s immune system to identify and destroy these cells.

### 2.2 Overview of the notch signaling pathway

The Notch signaling pathway was first discovered in mutant fruit flies, where the notched wing phenotype was caused by mutations in this gene (2), leading to the gene being named Notch. It was subsequently isolated in 1983 (3). The pathway is widely present in both invertebrates and vertebrates and is highly conserved throughout the evolutionary process of various species. Research has shown that the Notch signaling pathway is prevalent in multicellular organisms, facilitating intercellular communication via receptor-ligand interactions and significantly influencing cell differentiation, proliferation, apoptosis, and cell cycle regulation. The Notch signaling pathway plays a crucial regulatory role in multicellular animal development, tissue renewal, cellular homeostasis, and the pathogenesis of various diseases (4). The Notch signaling pathway’s dysregulation or loss is implicated in various human diseases, including developmental syndromes, adult disorders, and tumors (5).

The Notch signaling pathway is evolutionarily conserved and facilitates direct signal transmission between cell membrane receptors and nuclear effector molecules without intermediates. In contrast to traditional signaling pathways like those involving G protein-coupled receptors and enzyme-linked receptors, the Notch signaling pathway uniquely requires the receptor to undergo three cleavages for signal transmission to the nucleus. The Notch signaling pathway includes Notch receptors and ligands, intracellular effectors like CSL (CBF-1/Suppressor of Hairless/Lag-1) DNA-binding protein, downstream target genes such as the Hes (hairy and enhancer of split) and Hey (hairy and enhancer-of-split related with YRPW motif) gene families, along with regulatory molecules like Fringe, Numb, Deltex, and Mastermind (6). In mammals, four Notch receptors (Notch1-4) are encoded by distinct genes, with Notch1–3 linked to human diseases. The classical ligands include DLL1, DLL3, DLL4, and Jagged1-2 (Jag1, Jag2) (5).

The Notch signaling pathway comprises two types: the classical and non-canonical signal transduction pathways. The classical Notch signaling pathway begins when the Notch receptor binds to its ligand, leading to its cleavage by the -secretase complex and the subsequent release of the Notch intracellular domain (NICD). NICD translocates to the nucleus, engages with the CSL co-repressor complex, and activates transcription of downstream target genes. Research indicates that the Notch signaling pathway can operate independently of ligands and CSL via the non-canonical pathway, which bypasses -secretase cleavage and potentially influences cellular functions through alternative mechanisms (7–9). In vertebrates, atypical activation of Notch targets primarily occurs in lineage-restricted progenitor cells, specific differentiation pathways, and tumorigenesis (10).Research has shown that Notch influences the Wnt/β-catenin, JAK/STAT, PI3K/AKT, and post-translational NF-κB signaling pathways, thereby playing a role in its unique biological functions. In human mammary epithelial cells, Notch3 signaling atypically regulates the expression of the Wnt receptor FZD7 via the Notch pathway (11–14). This unique Notch signaling activates IL-6/JAK/STAT pathways in breast cancer cells and is modulated by IKKα/IKKβ within the NF-κB signaling cascade (15). Furthermore, The study revealed that atypical Notch signaling interacts with PTEN-induced kinase 1 (PINK1), influencing mitochondrial function and activating the mTORC2/AKT signaling pathway, which supports the maintenance of brain tumor stem cells (16). Perumalsamy et al. (17) discovered a novel Notch-mediated signaling pathway independent of CBF1/RBPJ, where NICD interacts with the mTOR-Rictor complex to activate AKT/PKB, thereby regulating mammalian cell survival.

The Notch signaling pathway’s activation and function are crucial for various cellular processes. This pathway is initiated when Notch receptors interact with specific ligands, leading to a series of proteolytic cleavages. These cleavages release the Notch intracellular domain, which translocates to the nucleus to regulate gene expression. The pathway plays a significant role in cell differentiation, proliferation, and apoptosis, highlighting its importance in developmental and physiological contexts.

### 2.3 Notch signaling pathway activation

The Notch receptor is a single-pass transmembrane heterodimer composed of the Notch extracellular domain (NECD), transmembrane domain (TMD), and Notch intracellular domain (NICD) (18). The NECD (N-terminus) of the Notch receptor comprises 29–36 epidermal growth factor-like repeats (EGF-R) and is followed by a negative regulatory region (NRR) (19, 20). The NRR domain consists of three cysteine-rich Lin12-Notch repeats (LNR) and a heterodimer essential for the cleavage site (21–23). In the traditional Notch signaling pathway, the Notch receptor is initially transported to the endoplasmic reticulum as a single-chain precursor. The EGF-like domain of the Notch receptor is glycosylated within the endoplasmic reticulum (24, 25). The glycosylated Notch precursor is transported to the Golgi apparatus, where furin-like proteases cleave it (S1 cleavage), producing NECD and TMD fragments (26, 27). These fragments bind via Ca^2+^-dependent non-covalent interactions, forming the mature heterodimeric Notch receptor. The mature Notch receptor, as a type I transmembrane protein, is transported to the cell membrane (28). The Notch heterodimeric transmembrane receptor binds to the transmembrane ligand on neighboring cells at the cell membrane, triggering the Notch signaling cascade. In the absence of ligands, the NRR domain can prevent receptor activation. Binding of the Notch signaling receptor to the Notch ligand on neighboring cells induces a conformational change in the receptor. After ubiquitination by Neur or Mib, the NRR domain is expanded, and the Notch receptor extracellular domain is hydrolyzed and dissociated under the catalysis of a disintegrin and metalloproteinase (ADAM), particularly ADAM 10 or ADAM 17 (29, 30). The S2 site exposure and subsequent cleavage result in the formation of the Notch extracellular truncation (NEXT) from the remaining portion of the Notch receptor. NEXT can be further cleaved by γ-secretase at the S3 site in the transmembrane region to produce NICD (31, 32). Subsequently, NICD enters the cytoplasm or translocates to the nucleus, Interacting with other signaling pathways. NICD enters the nucleus and binds to the transcription factor CSL (also known as RBPJ) to regulate gene transcription (33, 34). Upon binding to CSL, NICD changes CSL’s conformation from a transcriptional repressor to an activator and recruits the coactivator mastermind-like (MAML) to form a NICD/MAML/CSL ternary complex (35). This complex also triggers the expression of downstream target genes like Hes and Hey (36). These genes are transcriptional repressors that significantly influence cell differentiation and proliferation.

## 3 Functions of the notch signaling pathway

The Notch signaling pathway is essential for preserving cancer cells’ stem cell-like properties, increasing invasiveness, and fostering chemoresistance. This pathway can function as both an oncogene and a tumor suppressor across different cancer types. Dysregulation of the Notch signaling pathway can facilitate epithelial-mesenchymal transition (EMT) and angiogenesis in malignant tumors, processes that are intimately linked to cancer proliferation, invasion, and metastasis (37, 38). The Notch signaling pathway’s abnormal activation is linked to the emergence of chemoresistance in multiple tissue cancers. The Notch signaling pathway is crucial in regulating cancer stem cell formation and epithelial-mesenchymal transition (EMT), potentially providing new strategies to overcome cancer cell resistance in tumor therapy.

The Notch signaling pathway has garnered significant interest regarding its role in cancer energy metabolism. For example, oncogenic Notch signaling promotes T-ALL cell proliferation through Asb2-mediated NF-κB (39). The Notch signaling pathway mediated by DLL1 is also involved in the acquisition of stem cell-like characteristics in esophageal adenocarcinoma (40). The Notch signaling pathway occurs through the binding of a transmembrane ligand on one cell with the Notch receptor paralog on an adjacent cell, and its important target genes include the HES gene family, the proto-oncogene MYC, and CDKN1A (41). This pathway is involved in regulating cell proliferation, apoptosis, differentiation, and EMT, playing an essential role in the development, invasion, and metastasis of tumors.

### 3.1 The notch signaling pathway plays a crucial role in tumor development

The association between the Notch signaling pathway and tumor development was initially identified in human T-cell acute lymphoblastic leukemia (T-ALL) (42). Recent research highlights the Notch pathway’s significant role in both biological development and tumor occurrence and progression. Research indicates that the Notch signaling pathway is abnormally expressed in the development of various cancers (**Table 1**).

**Table 1:** NOTCH signaling in cancers.

Cancer type	Involved NOTCH components	Relevant evidence	Ref.
T-cell acute lymphoblastic leukemia	NOTCH1, NOTCH3	Transplanted hematopoietic progenitor cells with activation of Notch1 signaling in murine models can develop T-ALL; Activating mutations of NOTCH3 without NOTCH1 has also been found in several T-ALLs.	(115, 116)
Breastcancer	NOTCH1, NOTCH4, JAG1	Upregulation of non-mutated NOTCH1 and JAG1 is associated with poorprognosis of BC; The mutations of Notch1 and Notch4 mediated by the mouse mammary tumor virus can promote epithelial mammary tumorigenesis;	(117, 118)
Squamouscellcancers	NOTCH1-3	Inactivated NOTCH1–3 were detected in SCCs pecimens; The genomic aberrations in NOTCH1 induced by mutagenic agent could cause an increasing tumor burden in SCCs;	(119–121)

In oncology, the Notch signaling pathway has a highly intricate role, as it can either encourage or suppress the initiation and progression of various tumors, potentially having different effects at different stages of tumor growth. Due to the complexity and diversity of its regulatory mechanisms, the Notch signaling pathway has become an important potential target for the study and treatment of various diseases. In cervical cancer development, high Notch signaling expression inhibits cell growth, whereas low expression decreases apoptosis. Inhibiting the Notch signaling pathway expression induces apoptosis in pancreatic cancer cells (43). In non-small cell lung cancer (NSCLC), Notch1 and Notch2 function mainly as oncogenes, while Notch3 facilitates NSCLC development and progression. Although the role of Notch4 in NSCLC remains unclear, evidence indicates it may contribute to tumor progression. In breast cancer, Notch1 enhances cancer cell proliferation and sustains cancer stem cell activity, whereas Notch3 suppresses epithelial-mesenchymal transition (EMT) by activating glycogen synthase kinase 3 beta (GSK3β) expression (44). In triple-negative breast cancer (TNBC), the transcriptional repressor B-cell lymphoma 6 (BCL6) influences the tumor stem cell (TSC) compartment by modulating the Notch signaling pathway, thereby impacting the maintenance and expansion of TSC characteristics (45). Knocking out the Notch1 gene in mouse skin leads to epidermal hyperplasia and impaired cell differentiation, resulting in cell cancer (46). In various types of glioma, receptors such as Notch1, ligands Dll1, and Jagged1 in the Notch signaling pathway are all abnormally overexpressed (47). In addition to these roles, the Notch signaling pathway also plays important roles in other aspects. In a mouse ureter model, the Notch signaling pathway facilitates the differentiation of visceral smooth muscle cells (48). In a mouse brain injury model, Notch1 activation preserves blood-brain barrier integrity by supporting endothelial mitochondrial function (16). In a mouse polycystic ovary syndrome (PCOS) model, endoplasmic reticulum stress regulates the expansion of the cumulus oocyte complex (COC) by activating Notch2 (49).

### 3.2 The influence of notch signaling on cancer metabolism

The Notch signaling pathway is essential for intercellular communication, transmitting vital information in normal cells and playing a pivotal role in cancer development (50). The role of Notch signaling in tumor development and progression varies, being either oncogenic or tumor-suppressive, depending on the specific cell type and microenvironment, although its aberrant activation is linked to various cancers (51). In T-ALL, gain-of-function mutations in Notch1 drive tumorigenesis and disease progression by dual activation of the MYC proto-oncogene and the NF-κB signaling pathway (52). Similarly, in ErbB2-negative breast cancer models, Notch3 exerts oncogenic effects through the canonical CSL-dependent transcriptional regulatory mechanism (53). In contrast, in cutaneous squamous cell carcinoma (cSCC), Notch1 exhibits a tumor-suppressive role, inhibiting tumor initiation by maintaining epidermal stem cell homeostasis and promoting terminal differentiation (21). Together, these studies highlight the complex and finely tuned regulatory network of the Notch signaling pathway in cancer development and progression (**Table 2**).

**Table 2:** The relationship between the Notch signaling pathway and related diseases.

Type of disease	Point of application	Mechanism of action
Congenital scoliosis (CS)	Mutations in Notch Signaling Pathway-Related Genes (HES7, LFNG, MESP2, DLL3)	Gene Mutations Lead to Abnormal Spinal Development
Alagille Syndrome(ALGS)	JAG1 gene mutation	Mutations in the JAG1 gene lead to biliary ductal dysgenesis, thereby affecting the liver and other multiple systems.
Tetralogy of Fallot(TOF)	Variants in the 3′ untranslated region (3′UTR) of the NOTCH1 and JAG1 genes.	Genetic variants impact cardiac development, leading to abnormalities in the ventricular outflow tract.
Non-alcoholic Fatty Liver Disease(NAFLD)Non-alcoholic Fatty Liver Disease(NASH	Notch1/Notch2 Elevated expression of Notch1/Notch2	Aberrant activation of the Notch signaling pathway promotes lipogenesis and hepatic fibrosis.
Diabetes mellitus	Inhibition of the Notch signaling pathway	Inhibition of the Notch signaling pathway promotes the differentiation of pancreatic cells into β-cells, thereby improving glucose homeostasis.
Pancreatic Ductal Adenocarcinoma(PDAC)	Activation of the Notch signaling pathway	Synergizes with Ras to promote oncogenesis
T-cell acute lymphoblastic leukemia(T-ALL)	Gain-of-function mutations in Notch1	Elevated expression of Notch1 drives cell proliferation and oncogenesis.

In the reprogramming of cancer metabolism and the regulation of the tumor microenvironment, the Notch signaling pathway demonstrates its key role in balancing oncogenic and tumor-suppressive effects. The complexity of this pathway lies in its ability to exert entirely different functions in various biological contexts, making it a highly promising target in cancer therapy.

### 3.3 Metabolic reprogramming regulation

Metabolic reprogramming is essential in the development and progression of diseases such as cancer, diabetes, cardiovascular, and neurodegenerative disorders. It refers to the phenomenon where cellular metabolic pathways change under different physiological or pathological conditions, such as cancer, inflammation, infection, or cell differentiation. This metabolic shift involves multiple aspects, including energy production, biosynthesis, and cellular signaling, to adapt to changes in cellular function. Metabolic reprogramming is a hallmark of cancer, encompassing changes in the metabolism of glucose, fatty acids, and amino acids (**Table 3**).

**Table 3:** Types of metabolic reprogramming regulated by the notch signaling pathway.

Category	Primary mechanisms
Associated with glucose catabolism	Anaerobic glycolysis and aerobic oxidation
Associated with fatty acid catabolism	Fatty acid oxidation (β-oxidation)
Associated with amino acid catabolism	Protein degradation, amino acid deamination, and urea cycle

#### 3.3.1 Notch pathway and glucose metabolism

Glucose metabolism primarily occurs through two pathways: anaerobic and aerobic oxidation. Anaerobic oxidation, also known as glycolysis, is the process by which glucose is converted into lactate, while aerobic oxidation involves the complete oxidation of glucose in the presence of oxygen, ultimately producing water and carbon dioxide. Aerobic glycolysis supplies energy and facilitates the synthetic metabolic growth of cancer cells.

**Figure 1:**
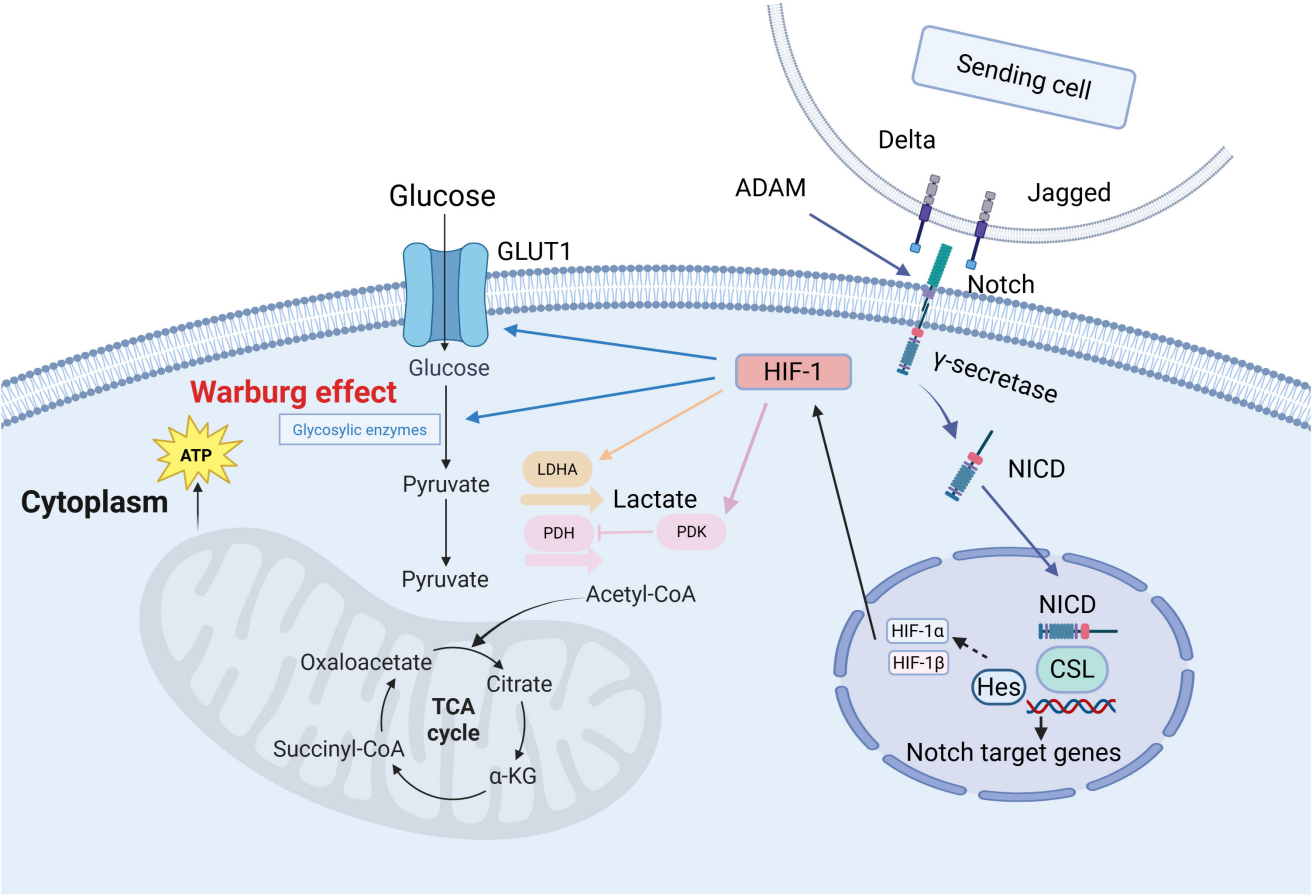
The Warburg effect in cancer cells (132). In the Notch signaling pathway, Delta/Jagged ligands bind to Notch receptors, which undergo sequential cleavage by ADAM and γ-secretase to release the Notch intracellular domain (NICD). NICD translocates into the nucleus and binds to the CSL transcription factor, activating target genes such as Hes to regulate cell fate decisions. Under hypoxic conditions, HIF-1α forms a heterodimeric complex with HIF-1β. This complex then recruits transcriptional co-activators to upregulate glycolytic genes—such as GLUT1 and PDK1—which promotes glucose uptake and glycolysis. These coordinated mechanisms demonstrate how Notch and HIF-1 pathways synergistically drive metabolic reprogramming to meet cellular energy demands.

The Notch signaling pathway is pivotal in the glycolysis phase of cancer cell metabolic reprogramming. This process, known as the Warburg effect (54), also generates substantial lactate production. This phenomenon was first discovered by German physiologist and Nobel laureate Otto Warburg (**Figure 1**). Excessive glucose intake can increase glycolytic intermediates, which subsequently enhances pentose phosphate pathway (PPP) activity (55). In short, the Warburg effect describes the abnormal metabolic activity observed in cancer cells, and this concept is continually being enriched and deepened with the advancement of science.

Research has demonstrated that in gastric cancer cells, activation of the Notch pathway significantly enhances glycolytic activity by upregulating downstream genes such as c-Myc, promoting the synthesis of a series of key glycolytic enzymes, including LDHA, PFKB3, PKM2, PGK1, HK2, GLUT1, ALDOA, PEPCK, and GLUT3 (56). This process relies on the synergistic action of the transcriptional coactivator TAZ and the p300/CBP-associated factor (pCAF). Notch1, as a critical member of this pathway, has been confirmed to play a regulatory role in various cell types. In human adipose-derived mesenchymal stem cells, activated Notch1 signaling elevates nuclear p65 levels, upregulates glycolytic factors such as GLUT3, and thereby promotes glycolysis (57–59). Notch1 signaling cooperates with the transcription factor FoxO1 to induce insulin resistance in hepatocytes of diabetic mouse models and to upregulate glucose-6-phosphatase expression, thereby disrupting hepatic glucose homeostasis (60). Notch3 activates mTORC1 via a non−canonical pathway, stabilizing hypoxia−inducible factor HIF−1; under normoxic conditions, stabilized HIF−1 upregulates glycolytic enzymes (GLUT1, HK2, PDK1) and suppresses mitochondrial oxidative phosphorylation, promoting a Warburg−like metabolic shift. In breast cancer—particularly triple−negative breast cancer—the Notch3/mTOR/HIF−1 signaling axis enhances glycolysis and thereby supports cancer stem cell survival and chemoresistance (61).

#### 3.3.2 Notch pathway and fatty acid metabolism

Lipids, or fats, consist of molecules such as triglycerides, phospholipids, sphingolipids, cholesterol, and cholesteryl esters. Among these lipids, fatty acids (FAs) are the core components, which not only constitute lipids but can also exist independently. The metabolism of FAs involves their uptake, synthesis, oxidation, transformation, and storage. The sources of FAs are divided into exogenous and endogenous. Cells uptake fatty acids from their environment via transport proteins on the plasma membrane, including fatty acid translocase (FAT), fatty acid transport proteins (FATPs/SLC27), and plasma membrane fatty acid-binding protein (FABP). Endogenous fatty acids are synthesized from acetyl-CoA in the cytoplasm, which is derived from glycolysis, the tricarboxylic acid cycle, or the pentose phosphate pathway.

**Figure 2:**
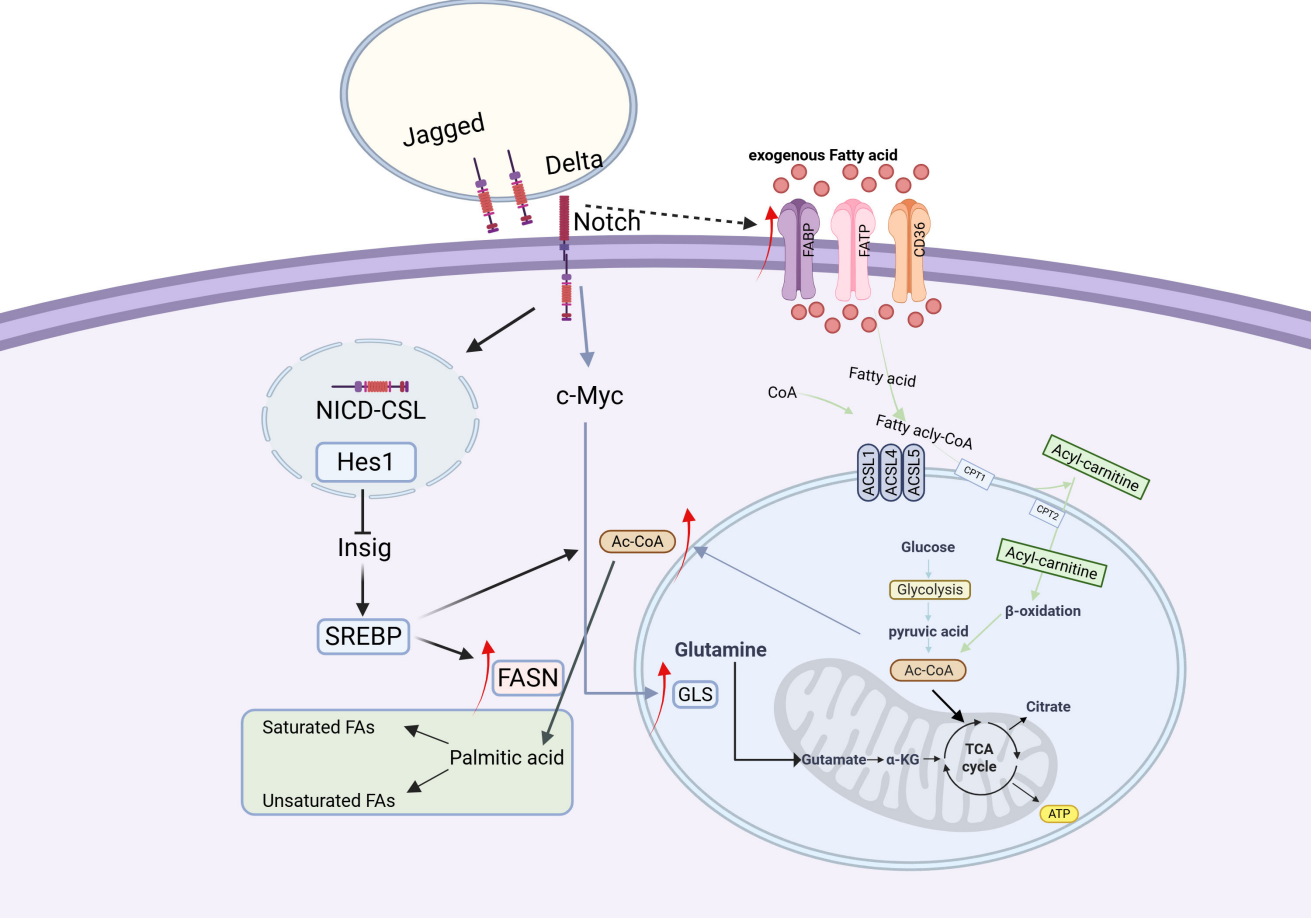
Fatty acid metabolism in cancer cells (133). In cancer cells, fatty acid (FA) metabolism is markedly enhanced, characterized by increased exogenous FA uptake and activated *de novo* lipogenesis. Acetyl-CoA serves as a pivotal metabolic hub, playing a central role in FA metabolism. Upon binding of Delta/Jagged ligands to Notch receptors, proteolytic cleavage releases the Notch intracellular domain (NICD). NICD translocates into the nucleus and forms a complex with the CSL transcription factor, which activates Hes1 expression, subsequently inhibits Insig protein, and ultimately leads to activation of the SREBP transcription factor. The activated SREBP upregulates the expression of fatty acid synthase (FASN) and promotes acetyl-CoA production. Concurrently, NICD enhances glutaminase (GLS) expression through c-Myc activation, thereby stimulating glutamine metabolism. Furthermore, the Notch signaling pathway indirectly activates fatty acid-binding proteins (FABPs), further driving the progression of fatty acid metabolism.

Fatty acid oxidation (FAO), or β-oxidation, is a vital mitochondrial process integral to the energy metabolism and stress response in cancer cells. Key enzymes such as carnitine palmitoyltransferase I (CPT 1) are potential targets for cancer therapy (62). During FAO, FAs are first converted to acyl-CoA, then bind to carnitine to form acylcarnitine, a step mediated by carnitine-acylcarnitine translocase (CACT). Acylcarnitine is then converted back to acyl-CoA by carnitine palmitoyltransferase II (CPT 2) and continues to participate in the oxidation process (**Figure 2**).

Fatty acid synthesis *de novo* depends on glucose metabolism for its carbon source, with citrate serving as a crucial intermediate. Enzymes including ATP citrate lyase (ACLY), acetyl-CoA carboxylase (ACC), fatty acid synthase (FAS), and acyl-CoA synthetase facilitate the conversion of citrate-derived carbon into biologically active fatty acids. The activity of these enzymes significantly impacts the biological functions of cancer cells, and inhibiting their activity may be beneficial for cancer treatment.

The Notch signaling pathway orchestrates lipid metabolic homeostasis through multi-level regulatory mechanisms. This pathway promotes hepatic fatty acid synthesis by maintaining the stability of mTORC1 (mechanistic target of rapamycin complex 1) (63). Studies in liver-specific Rbpj knockout mice demonstrate that reduced mTORC1 stability significantly decreases hepatic triglyceride accumulation and downregulates expression of key lipogenic enzymes FASN and ACC1 (63). Concurrently, Notch1 inhibition enhances hepatic fatty acid oxidation capacity, as evidenced by upregulated expression of β-oxidation-related genes including CPT1, ACOX1 and PPAR (64). Mechanistic investigations reveal that the Notch1 signaling pathway exhibits dual regulatory effects: it suppresses lipogenesis by downregulating SREBP-1c while simultaneously promoting fatty acid oxidation through increased expression of CPT1 and ACAAOX1 (65). In renal systems, Notch signaling negatively regulates the expression of the metabolic master regulator PGC-1 via its downstream effector Hes1 (66–69), whereas Hes1 can indirectly stimulate lipogenesis by inhibiting the activity of transcription factors (70). In hepatic tissue, Notch signaling directly modulates the expression of fatty acid transport proteins via nuclear-translocated NICD-FoxO1 complexes that bind to the FABP4 gene promoter region (71, 72). Notch signaling exhibits tissue-specific regulatory patterns across different cancer types: in ovarian cancer, it promotes lipogenesis through activation of the SREBP-1/FASN axis (73), in triple-negative breast cancer, it impairs fatty acid oxidation via Hes1-mediated suppression of CPT1A expression (74), while in hepatocellular carcinoma, the HIF-1α-mediated inhibition of FAO can be reversed through combination targeted therapies (75).

#### 3.3.3 Notch pathway and amino acid metabolism

Amino acid metabolism and catabolism mainly include protein degradation, amino acid deamination, and the urea cycle. Amino acids play significant physiological roles in the body, such as protein synthesis, signal transduction, and gene expression regulation. Glutamine metabolism is essential in tumor cells. Activation of the Notch signaling pathway enhances glutaminase expression, boosting glutamine metabolism to supply nitrogen and energy to tumor cells.

In acute T-cell lymphoblastic leukemia (T-ALL), inhibiting the Notch1 signaling pathway influences glutamine catabolism (76).Activated Notch1 signaling influences the growth and metabolism of leukemia cells through the MYC protein (77).The MYC protein can upregulate the expression of branched-chain aminotransferase 1 (BCAT1), promoting the catabolism of branched-chain amino acids (BCAA) (78–80); and it can promote the translation of glutaminase 1 (GLS1) by inhibiting miR-23a/b, thereby increasing glutamine catabolism (81, 82).

Notch signaling also participates in regulating the activity of mTORC1 (83, 84) and driving the uptake of glutamine (85), which is crucial for maintaining the transport of leucine and glutamine during amino acid metabolism (86) (**Table 4**). These findings highlight the diverse functions of the Notch signaling pathway in amino acid metabolism, offering novel insights for treating related diseases.

**Table 4:** Notch and metabolic pathways.

Metabolic pathways	Interaction with the notch pathway	Tumor types	Ref.
mTOR	Notch activates the PI3K/AKT/mTOR pathway to promote tumor growth. Notch1 activates the mTORC1 pathway. The mTOR pathway cooperates with Notch signaling and metabolic reprogramming to maintain breast cancer stem cell characteristics and promote therapy resistance.	Breast cancer, T-ALL, and breast cancer stem cells (BCSCs).	(122, 123)
HIF-1α	Notch stabilizes HIF-1α to promote the Warburg effect. Hypoxia enhances Notch signaling (upregulating DLL4). Notch and HIF-1α cooperatively regulate EMT progression and metabolic reprogramming in breast cancer. Notch1 and HIF-1α show synergistic co-expression in breast cancer.	Glioblastoma (GBM), Renal cell carcinoma (RCC), Breast cancer	(108, 124, 125)
MYC	Notch1 directly activates MYC transcription. MYC inhibits the pro-apoptotic function of Notch2. MYC-activated NOTCH signaling mediates neuroendocrine differentiation in SCLC.	T-ALL, Breast cancer, NSCLC、SCLC	(126, 127)
Wnt/β-catenin	Notch and Wnt competitively regulate stem cell differentiation. β-catenin inhibits Notch1.	Colorectal cancer (CRC), HCC	(128–130)
p53	p53 inhibits Notch1 expression and impedes cell differentiation. Notch1 reduces proteasomal degradation of p53 by suppressing the Akt/Hdm2 pathway, upregulates p53, and enhances apoptosis sensitivity in HCC cells.	cervical cancer, HCC	(109, 131)

### 3.4 The role of notch signaling pathway in the tumor microenvironment

The tumor microenvironment (TME) primarily comprises blood vessels, immune cells, fibroblasts, and the extracellular matrix (87), and is essential for tumor initiation and progression. Tumor progression, treatment resistance, invasion, and metastasis are linked to the interaction between tumor cells and the tumor microenvironment (TME) (88). The Notch signaling pathway plays a crucial regulatory role in diverse cell types within the tumor microenvironment, such as cancer-associated fibroblasts (CAFs), myeloid-derived suppressor cells (MDSCs), regulatory T cells (Tregs), natural killer (NK) cells, and tumor-associated macrophages (TAMs) (**Table 5**).

**Table 5:** Regulation of multiple cell types in the tumor microenvironment by the notch signaling pathway.

Cell type	Regulatory mechanisms	Specific functions
Fibroblasts	Activation of the Notch signaling pathway	Promotes the activation of cancer-associated fibroblasts (CAFs), secretion of extracellular matrix components, and remodeling of the tumor microenvironment.
Endothelial cells	The Dll4/Notch1 signaling pathway	Through interaction with tumor cells, it inhibits tumor cell proliferation and participates in tumor angiogenesis.
Endothelial cells	Interplay between Notch signaling and immune cells	Regulates the polarization of tumor-associated macrophages (TAMs) to influence the immunosuppressive microenvironment; affects the activity and function of T cells, which may promote immune evasion.
Stem cells	Notch signaling sustains stem cell properties.	Sustains the self-renewal and differentiation capacity of cancer stem cells, thereby promoting tumor recurrence and drug resistance.
Tumor cells	Activation or inhibition of the Notch signaling pathway	Promotes tumor cell proliferation, survival, and inhibits apoptosis; regulates epithelial-mesenchymal transition (EMT), thereby enhancing the capacity for invasion and metastasis; sustains the stemness properties of cancer cells, augmenting tumor aggressiveness.

#### 3.4.1 Regulation of CAFs by notch signaling pathway

CAFs play a significant role in maintaining an ideal microenvironment for tumor cell survival and proliferation and promoting angiogenesis (89). These cells can secrete interleukin-6 (IL-6), which in turn activates the Notch signaling pathway, enhancing the stem cell-like characteristics of liver cancer cells and promoting the progression of liver cancer (90). In breast cancer, CAFs secrete IL-6 to activate Notch signaling in cancer cells, which also mediates the effects of estrogen G protein-coupled receptor (GPER) signaling in both cancer cells and CAFs (91, 92). Additionally, Jagged1 from CAFs interacts with Notch3 in tumor cells to regulate tumor resistance. In liver cancer, CAFs induce the expression of Notch3, promoting the proliferation of tumor stem cells. Additionally, the Notch3 expression induced by liver cancer CAFs helps promote the proliferation of tumor stem cells (93, 94).

#### 3.4.2 Notch signaling pathway regulates MDSCs

During tumor immune escape, myeloid-derived suppressor cells (MDSCs) play a key role. Tumor cells attract MDSCs to the target organ by secreting cytokines or chemokines. In this process, MDSCs exert their inhibitory function by secreting arginase-1, inducible nitric oxide synthase, and reactive oxygen species, which can effectively inhibit the proliferation of CD4^+^ and CD8^+^ T cells. MDSCs secrete interleukin-10 and TGF-β, enhancing regulatory T cell (Treg) recruitment and contributing to an immunosuppressive environment (95).

#### 3.4.3 Regulation of tregs by notch signaling pathway

Regulatory T cells (Tregs), a subset of CD4^+^ T cells, are crucial in tumor development due to their immunosuppressive function. The Notch signaling pathway is essential for FOXP3 transcription regulation and Treg differentiation (96). Research indicates a negative correlation between mRNA expression levels of the Notch signaling pathway and both the quantity of Tregs and FOXP3 mRNA expression in tumor tissues. This indicates that the downregulation of Notch signaling may promote the infiltration of Tregs into the tumor microenvironment, especially in triple-negative/basal-like breast cancer, where this effect is particularly significant (97).

#### 3.4.4 Regulation of NK cells by the notch signaling pathway

As critical effector cells of the innate immune system, natural killer (NK) cells directly eliminate tumor cells through cytotoxic activity. The Notch signaling pathway not only plays a pivotal role in regulating the development and functional maturation of NK cells but also establishes a unique immune evasion mechanism in cancer stem cells (CSCs) via the Notch3 receptor. Studies have demonstrated that Notch3 is the only member of the Notch receptor family confirmed to directly regulate the expression of programmed death-ligand 1 (PD-L1) (98). Specifically upregulated in breast CSCs, Notch3 significantly enhances PD-L1 expression by activating the mTOR signaling pathway and cooperatively modulating transcription factors such as c-Myc and Stat3 (99, 100). This regulatory mechanism is essential for maintaining CSC stemness, with Notch3 expression patterns closely associated with the phenotype of mammary luminal progenitor cells characterized by high clonogenic capacity and transient quiescence (101, 102). In HER2/neu–negative breast cancer, Notch3 promotes tumor cell proliferation by upregulating PD-L1 (103) and facilitates immune evasion by creating an immunosuppressive tumor microenvironment (53). Experimental evidence confirms that Notch3 knockout or inhibitor treatment significantly reduces PD-L1 expression and CSC stemness, while mTOR silencing completely abolishes Notch3-mediated PD-L1 regulation (93). These findings highlight the central role of the Notch3/mTOR/PD-L1 axis in sustaining the immunosuppressive properties of CSCs, providing a theoretical foundation for novel combination therapies. For instance, combining Notch3 inhibitors (e.g., Tarextumab) with anti-PD-L1 immune checkpoint blockade may overcome current immunotherapy resistance by simultaneously targeting CSC stemness and immune evasion mechanisms. Notably, elevated Notch3 expression may serve as a potential biomarker for predicting immunotherapy response, a discovery with particular clinical relevance in triple-negative breast cancer (TNBC).

#### 3.4.5 Regulation of TAMs by the notch signaling pathway

The Notch signaling pathway is essential for controlling the polarization of tumor-associated macrophages (TAMs), encouraging their conversion into the M1 type, which is an anti-tumor active phenotype. The Notch signaling pathway facilitates macrophage polarization to the M1 type by modulating downstream molecules miR-125a and miR-148a-3p expression (104). Additionally, the Notch signaling pathway also regulates the polarization of macrophages through its downstream SOCS3 molecule. Activation of the Notch signaling pathway induces macrophage polarization to the M1 type, boosting their anti-tumor capabilities and suppressing tumor growth (105).

### 3.5 The dual role of notch signaling in cancer pathogenesis

The Notch signaling pathway exhibits a complex dual role in cancer, with its dysregulation being closely associated with the pathogenesis of various malignancies. However, recent studies have revealed significant tumor type-dependent heterogeneity and metabolic crosstalk mechanisms. In terms of targeted therapies, γ-secretase inhibitors (GSIs) such as nirogacestat have demonstrated efficacy in desmoid tumor clinical trials but face dose-limiting gastrointestinal toxicity in solid tumors (106). Meanwhile, novel antibody-based agents (e.g., anti-DLL3/4 antibodies) and bispecific T-cell engagers (e.g., AMG 119) show promise in T-ALL and small cell lung cancer (SCLC), yet encounter challenges due to vascular toxicity caused by insufficient receptor/ligand selectivity (107). Metabolic studies demonstrate that Notch crosstalk with mTOR/HIF-1 can drive the Warburg effect and glutamine addiction (59, 108). Paradoxically, while Notch1 enhances TRAIL sensitivity in hepatocellular carcinoma by stabilizing p53 through Akt/Hdm2 inhibition (109), it cooperates with HIF-1 to promote epithelial-mesenchymal transition (EMT) in breast cancer (108). This context-dependent effect is particularly prominent in the tumor microenvironment. For instance, cancer-associated fibroblast (CAF)-derived Jagged1 activates Notch signaling in tumors, whereas hypoxia-induced crosstalk between Notch3 and the G-protein coupled estrogen receptor (GPER) can antagonize therapeutic responses in estrogen receptor-positive breast cancer. The RATIONALE-302 trial demonstrated significantly improved median overall survival in NOTCH1-mutated patients treated with tislelizumab compared to chemotherapy (18.4 months vs 5.3 months, HR = 0.35) (110). In desmoid tumors, the phase III clinical trial of γ-secretase inhibitor nirogacestat showed remarkable disease control (106). Notably, GSIs can effectively reverse paclitaxel resistance in triple-negative breast cancer by modulating the γ-secretase/Notch/PXR signaling axis, significantly enhancing chemosensitivity (111). Several GSIs, including DAPT, PF-03084014, and RO4929097, have entered clinical investigation and demonstrated efficacy in overcoming drug resistance across various malignancies, including prostate cancer (112–114) (**Table 6**).

**Table 6:** Clinical targeting of the notch pathway.

Target	Drug name	Conditions	Phase	Current states
γ-Secretase	RO4929097 (R4733)	Breast Cancer	I	Terminated
		Metastatic Pancreas Cancer	II	Completed
		Advanced Solid Tumors	I	Completed
	Nirogacestat (PF 03084014)	Desmoid Tumors/Aggressive Fibromatosis	II	Active, not recruiting
	AL101	Advanced solid tumors	I	Completed
		Advanced Breast Cancer	II	Terminated
		Advanced Cancer And Leukemia	I	Completed
DLL3	Rovalpituzumab tesirine (Rova-T)	Small cell lung cancer	III	Completed
	HPN-328	Small cell lung cancer	I/II	Terminated
DLL4	Enoticumab (REGN421)	Advanced Solid Malignan cies	I	Completed
	Demcizumab (OMP 21M18)	Non-Squamous Non Small Cell Lung Cancer	I	Completed
Notch 1	Brontictuzumab (OMP 52M51)	Solid Tumors	I	Completed
Notch 3	PF-06650808	Advanced solid tumors	I	Terminated
Notch 2/3	Tarextumab (OMP-59R5)	Pancreatic Cancer	I/II	Completed

## 4 Summary and future prospects

Targeting the Notch signaling pathway has demonstrated significant therapeutic potential in various malignancies, with current clinical development primarily focusing on three key strategies: 1) γ-secretase inhibitors (GSIs), including nirogacestat, DAPT, and PF-03084014; 2) Notch receptor/ligand-targeting antibodies, such as DLL3-targeted tarlatamab; and 3) synthetic Notch (synNotch) therapies. Clinical evidence indicates that NOTCH1 mutation status serves as a predictive biomarker for immunotherapy efficacy in esophageal squamous cell carcinoma (ESCC).

However, Notch-targeted therapies face several critical challenges: First, GSIs exhibit dose-limiting toxicities, including gastrointestinal effects, dermatologic adverse events, and potential impacts on growth and development, along with off-target effects that limit clinical utility. Second, the pathway demonstrates context-dependent oncogenic or tumor-suppressive functions (e.g., pro-tumorigenic in NSCLC versus tumor-suppressive in EBT), necessitating highly individualized treatment approaches. Third, complex resistance mechanisms involving crosstalk with key pathways like Wnt and PXR substantially complicate therapeutic outcomes. Fourth, while DLL3-targeted agents (e.g., Amgen’s tarlatamab) have achieved breakthroughs in small cell lung cancer treatment, drug resistance remains problematic, and biomarker selection systems require optimization Fifth, although natural compounds like quercetin show specific Notch pathway modulatory activity, their low bioavailability presents a significant challenge. To address these challenges, future research should prioritize the following: 1) the development of highly selective novel inhibitors, particularly those derived from optimized natural products; 2) the establishment of precision biomarker stratification systems for personalized therapy; 3) the investigation of combination strategies involving immune checkpoint inhibitors (e.g., PD-1 antibodies) and targeted therapies; 4) the enhancement of drug delivery efficiency through nanotechnology; 5) the formulation of spatiotemporal sequential treatment strategies to overcome tumor heterogeneity; and 6) the conduct of large-scale, multicenter clinical trials to thoroughly evaluate long-term efficacy and safety. These advancements will facilitate the translation of Notch-targeted therapies from the laboratory to clinical practice, thereby opening new avenues for precision oncology.
