# Supplementary material for: Notch signaling in cancer: metabolic reprogramming and therapeutic implications
Source: Front Immunol. 2025 Sep 19;16:1656370. doi: 10.3389/fimmu.2025.1656370 (PMC12491222; doi:10.3389/fimmu.2025.1656370)
Supplement: Supplementary file 1 [file DataSheet1.pdf]

Acronym	English name
ACC	acetyl-CoA carboxylase
ACLY	ATP citrate lyase
ADAM	a disintegrin and metalloproteinase
a-KG	α-ketoglutarate
ATP	Adenosine Triphosphate
Ac-CoA	Acetyl-CoA
ACOX1	Acyl-CoA Oxidase 1
ALGS	Alagille Syndrome
BCAA	branched-chain amino acids
BCAT1	branched-chain aminotransferase 1
BCL6	B-cell lymphoma 6
BCSCs	Breast Cancer Stem Cells
CACT	carnitine-acylcarnitine translocase
CAFs	cancer-associated fibroblasts
COC	Cumulus oocyte complex
CPT 1	carnitine palmitoyltransferase I
CPT 2	Carnitine palmitoyltransferase II
CSL	transcription factor (CBF-1/Suppressor of Hairless/Lag-1)
CS	Congenital Scoliosis
CSCs	Cancer Stem Cells
cSCC	Cutaneous Squamous Cell Carcinoma
CRC	Colorectal Cancer
DALYs	disability-adjusted life years
DLL1, DLL3, DLL4	Notch ligands（Delta-Like Ligand）
EGF-R	epidermal growth factor-like repeats
EMT	epithelial-mesenchymal transition
ESCC	Esophageal Squamous Cell Carcinoma
FABP	fatty acid-binding protein
FABP4	fatty acid binding protein 4
FAs	fatty acids
FAS	fatty acid synthase
FASN	Fatty Acid Synthase
FAT	fatty acid translocase
FATP	Fatty Acid Transport Protein
FAO	Fatty Acid Oxidation
FZD7	Frizzled Class Receptor 7
GBM	Glioblastoma
GLUT1	Glucose Transporter 1
GLUT3	Glucose Transporter 3

GSI	γ-Secretase Inhibitor
GLS1	translation of glutaminase 1
GOT2	glutamate-oxaloacetate transaminase 2
GPT2	glutamate-pyruvate transaminase 2
GPER	G protein-coupled receptor
GSK3β	Glycogen synthase kinase 3 beta
HAMSCs	human adipose-derived mesenchymal stem cells
Hes	hairy and enhancer of split
Hey	hairy and enhancer-of-split related with YRPW motif
HK2	Hexokinase 2
HCC	Hepatocellular Carcinoma
HIF-1α	Hypoxia-Inducible Factor 1-Alpha
HR	Hazard Ratio
IL-6	interleukin-6
Jag 1-2	Notch ligands （Jagged1-2）
LDHA	Lactate Dehydrogenase A
LNR	Lin12-Notch repeats
MAML	mastermind-like
MCT4	monocarboxylate transporter 4
MDSCs	myeloid-derived suppressor cells
MYC	Myelocytomatosis oncogene
mTOR	Mechanistic Target of Rapamycin
mTORC1	mTOR Complex 1
mTORC2	mTOR Complex 2
NAFLD	Non-Alcoholic Fatty Liver Disease
NASH	Non-Alcoholic Steatohepatitis
NF-κB	Nuclear Factor Kappa B
NECD	Notch extracellular domain
NEXT	Notch extracellular truncation
NICD	Notch intracellular domain
NK	natural killer
NRR	Negative control region
NSCLC	non-small cell lung cancer
Notch1-4	Notch receptors1-4
NTR	Negative Control Region
PCOS	Polycystic ovary syndrome
PDAC	Pancreatic Ductal Adenocarcinoma
PD-L1	programmed death-ligand 1

PFKB3	6-Phosphofructo-2-Kinase
PGC1α	Peroxisome Proliferator-Activated Receptor Gamma Coactivator 1-Alpha
PINK1	PTEN-induced kinase 1
PI3K	Phosphoinositide 3-Kinase
RBPJ	Recombination Signal Binding Protein for Immunoglobulin Kappa J Region
RCC	Renal Cell Carcinoma
SCLC	Small Cell Lung Cancer
SCC	Squamous Cell Carcinoma
SREBP	Sterol Regulatory Element-Binding Protein
synNotch	Synthetic Notch
T-ALL	T-cell acute lymphoblastic leukemia
TAMs	tumor-associated macrophages
TCA	tricarboxylic acid
TMD	transmembrane domain
TNBC	triple-negative breast cancer
Tregs	regulatory T cells
TSC	tumor stem cell
TGF-β	Transforming Growth Factor Beta
TME	Tumor Microenvironment
